# ESOPEC: prospective randomized controlled multicenter phase III trial comparing perioperative chemotherapy (FLOT protocol) to neoadjuvant chemoradiation (CROSS protocol) in patients with adenocarcinoma of the esophagus (NCT02509286)

**DOI:** 10.1186/s12885-016-2564-y

**Published:** 2016-07-19

**Authors:** Jens Hoeppner, Florian Lordick, Thomas Brunner, Torben Glatz, Peter Bronsert, Nadine Röthling, Claudia Schmoor, Dietmar Lorenz, Christian Ell, Ulrich T. Hopt, J. Rüdiger Siewert

**Affiliations:** Department of General and Visceral Surgery, Medical Center - University of Freiburg, Hugstetter Str. 55, 79106 Freiburg, Germany; University Cancer Center Leipzig (UCCL), University Medicine Leipzig, Leipzig, Germany; Department of Radiation Oncology, Medical Center - University of Freiburg, Freiburg, Germany; Institute for Surgical Pathology, Medical Center - University of Freiburg, Freiburg, Germany; Clinical Trials Center, Medical Center - University of Freiburg, Freiburg, Germany; Department of Surgery, Sana Medical Center Offenbach, Offenbach, Germany; Department of Gastroenterology, Sana Medical Center Offenbach, Offenbach, Germany; Medical Center, University of Freiburg, Freiburg, Germany

**Keywords:** Esophageal cancer, Adenocarcinoma, Perioperative chemotherapy, Neoadjuvant chemoradiation

## Abstract

**Background:**

Recent randomized controlled trials comparing neoadjuvant chemoradiation plus surgery or perioperative chemotherapy plus surgery with surgery alone showed significant survival benefits for combined modality treatment of patients with localized esophageal adenocarcinoma. However, head-to-head comparisons of neoadjuvant chemoradiation and perioperative chemotherapy applying contemporary treatment protocols are lacking. The present trial was initiated to obtain valid information whether neoadjuvant chemoradiation or perioperative chemotherapy yields better survival in the treatment of localized esophageal adenocarcinoma.

**Methods/design:**

The ESOPEC trial is an investigator-initiated multicenter prospective randomized controlled two-arm trial, comparing the efficacy of neoadjuvant chemoradiation (CROSS protocol: 41.4Gy plus carboplatin/paclitaxel) followed by surgery versus perioperative chemotherapy and surgery (FLOT protocol: 5-FU/leucovorin/oxaliplatin/docetaxel) for the curative treatment of localized esophageal adenocarcinoma. Patients with cT1cN + cM0 and cT2-4acNxcM0 esophageal and junctional adenocarcinoma are eligible. The trial aims to include 438 participants who are centrally randomized to one of the two treatment groups in a 1:1 ratio stratified by N-stage and study site. The primary endpoint of the trial is overall survival assessed with a minimum follow-up of 36 months. Secondary objectives are progression-free survival, recurrence-free survival, site of failure, postoperative morbidity and mortality, duration of hospitalization as well as quality of life.

**Discussion:**

The ESOPEC trial compares perioperative chemotherapy according to the FLOT protocol to neoadjuvant chemoradiation according to the CROSS protocol in multimodal treatment of non-metastasized recectable adenocarcinoma of the esophagus and the gastroesophageal junction. The goal of the trial is identify the superior protocol with regard to patient survival, treatment morbidity and quality of life.

**Trial registration:**

NCT02509286 (July 22, 2015)

**Electronic supplementary material:**

The online version of this article (doi:10.1186/s12885-016-2564-y) contains supplementary material, which is available to authorized users.

## Background

Esophageal adenocarcinoma is one of the most rapidly increasing tumor entities in the Western world. Traditional curative treatment of localized esophageal adenocarcinoma consisted of esophagectomy, achieving 3- and 5-year survival rates of 40 %/39 % (thoraco-abdominal esophagectomy) and 34 %/27 % (transhiatal esophagectomy) *p* = 0.12) [1].

In addition to surgical resection, different neoadjuvant/perioperative strategies have been successfully developed to improve survival. Neoadjuvant chemoradiation is increasingly being applied and has also been introduced into different national and international guidelines [2]. The randomized Neoadjuvant chemoradiation followed by surgery versus surgery alone for patients with adenocarcinoma or squamous cell carcinoma of the esophagus (CROSS) trial (366 patients, recruitment 2004–2008) showed an improvement of overall survival after neoadjuvant chemoradiation with 41.4 Grey (Gy) and concomitant weekly carboplatin area under the curve (AUC) 2 mg/ml/min and paclitaxel 50 mg/m^2^ and subsequent surgical resection compared to surgery alone (esophageal adenocarcinoma: 3-year survival 55 % vs. 46 % *p* = 0.049) [3]. A significant overall survival benefit with neoadjuvant chemoradiation and surgery compared to surgery alone for patients with esophageal adenocarcinoma was also confirmed by two systematic reviews [4, 5].

Beyond that, different protocols of perioperative chemotherapy (neoadjuvant + adjuvant) have been applied as an alternative to neoadjuvant chemoradiation [6–8]. However, proofs of a clear survival benefit for patients with esophageal adenocarcinoma are missing because successfully completed randomized controlled trials (RCT) comparing perioperative chemotherapy with neoadjuvant chemoradiation are missing thus far and indirect comparisons are based on pooled and more or less heterogeneous patient cohorts including esophageal adenocarcinoma and gastric cancer [6, 7]. With regard to perioperative chemotherapy, specifically two multicenter trials (MAGIC [6] and ACCORD-07 [7]) found a significant increase in overall survival with cisplatin-fluoropyrimidine-based perioperative chemotherapy plus surgery compared to surgery alone. These two RCTs were carried out between 1994 and 2003 and recruited 26 % [6] and 75 % [7], respectively of the patients with esophageal adenocarcinoma or junctional adenocarcinoma.

Besides the cisplatin-based protocols, oxaliplatin-based chemotherapy protocols (FLO/FLOT: 5-FU, leucovorin, oxaliplatin, +/− docetaxel) have been developed and have been shown to be safe and efficacious for the perioperative treatment of esophageal adenocarcinoma and gastric cancer [8, 9]. Encouraging results have been reported [8] for perioperative chemotherapy in patients with esophagogastric adenocarcinoma with 3-year survival rates of up to 72 % using the FLOT regimen. The FLOT4 RCT (NCT01216644) which is currently running with the epirubricine-cisplatin-5FU or capecitabine (ECF/ECX) regimen as a comparator, will give conclusive data on safety and efficacy of the FLOT regimen.

The findings of the mentioned publications on perioperative chemotherapy and neoadjuvant chemoradiation were confirmed in our own patients (*n* = 47) treated with perioperative chemotherapy with ECF (*n* = 17) or FLOT (*n* = 29) (3y/5y survival 68/63 %) or with neoadjuvant chemoradiation (*n* = 58) (36 or 45 Gy, cisplatin and 5-FU) (3y/5y survival 52/45 %) [10]. Apart from a single RCT which compared neoadjuvant chemoradiation with neoadjuvant chemotherapy (carried out between 2000–2005 and terminated prematurely due to poor accrual without showing significant differences concerning survival), this is the only comparative analysis on neoadjuvant chemoradiation versus perioperative chemotherapy in patients with esophageal adenocarcinoma [11].

### Rationale for the trial

According to the given evidence, a survival benefit of perioperative chemotherapy over neoadjuvant chemoradiation for patients with esophageal adenocarcinoma has not been proven in any RCT. Data supporting the value of perioperative chemotherapy have all been obtained in studies of mixed patient cohorts with esophageal adenocarcinoma and gastric cancer. Due to relevant differences of histologic and molecular subtype distribution, different response rates to perioperative chemotherapy and survival rates according to anatomic location, there is a clear need to obtain evidence concerning the value of perioperative chemotherapy for esophageal adenocarcinoma and compare its effectiveness with neoadjuvant chemoradiation [8, 12–14]. As nowadays perioperative chemotherapy is extensively and successfully applied in clinical practice in patients with esophageal adenocarcinoma, there is an obvious need to obtain evidence from a multicenter RCT.

Secondly, confirmation of the survival outcomes reported in the recently published CROSS study assessing neoadjuvant chemoradiation [3] merit validation in a separate RCT conducted exclusively in esophageal adenocarcinoma.

## Methods/design

### Design

**Fig. 1:**
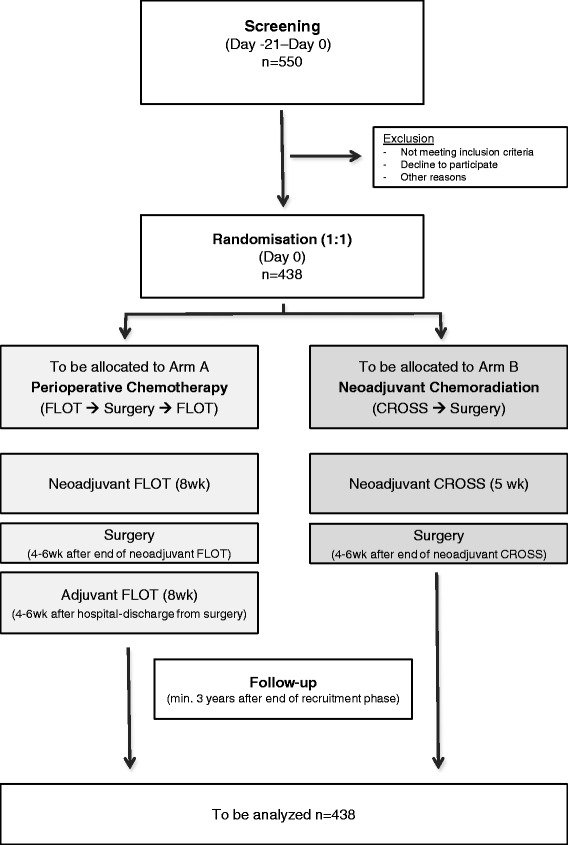
Trial diagram of the ESOPEC trial

The ESOPEC trial is a two-arm randomized phase III study in which patients are randomized to either perioperative chemotherapy according to the FLOT regimen (4 preoperative and 4 postoperative cycles of FLOT) or to neoadjuvant chemoradiation according to the CROSS regimen (41.1Gy plus carboplatin/paclitaxel) (Fig. 1). The primary objective of this phase III trial is to investigate whether perioperative chemotherapy improves overall survival compared to neoadjuvant chemoradiation. To achieve this objective, 438 patients will be recruited for the trial. Randomization is performed in blocks concealed from the investigator in a ratio of 1:1 with N stage (cN0/cN+) and recruitment site as stratification factors. Written informed consent is obtained from all patients prior to participation in the trial. Patient recruitment is taking place at 16 trial centers in Germany. All participating centers are highly experienced in gastrointestinal oncology and esophageal surgery and are performing at least 20 combined modality treatments of patients with localized esophageal adenocarcinoma per year in their center.

### Target population

Patients of both genders with histologically-proven adenocarcinoma of the esophagus (according to Union internationale contre le cancer (UICC) TNM7) [15] are enrolled in this trial; eligible patients must have non-metastatic disease. Patients are only allowed to enter the trial if they provide their written informed consent and if the responsible investigator has verified that all selection criteria are met.

### Inclusion criteria

Eligible patients must meet all of the following criteria:

1. Histologically-proven adenocarcinoma of the esophagus according to the Un UICC TNM7 definition [15]. Tumors of the esophagus and tumors of which the epicenter is within 5 cm of the esophagogastric junction and also extend into the esophagus are both eligible for inclusion in the trial in the case of adenocarcinomatous histology. Translated to Siewerts classification of esophagogastric adenocarcinoma (AEG), all Type AEG 1 are eligible. Type AEG 2 and Type AEG 3 are eligible in case of esophageal infiltration.
2. Pre-treatment stage cT1N+ M0 or cT2-4a N0/N+, M0 (In case of stage cT4a, curative resectability has to be explicitly verified by the local surgical investigator prior to randomization).
3. Age ≥ 18 years
4. No prior abdominal or thoracic radiotherapy
5. Eastern Cooperative Oncology Group (ECOG) performance status 0–2
6. Adequate cardiac function. Patients with a cardiac history should have a cardiology consultation and should have a left ventricular ejection fraction > 50 % (determined by echocardiography)
7. Adequate respiratory function (pulmonary function tests only necessary in symptomatic patients)
8. Adequate bone marrow function (White Blood Cells >3x10^9/l; Hemoglobin > 9 g/dl; platelets >100x10^9/l)
9. Adequate renal function (Glomerular filtration rate >60 ml/min) and
10. Adequate liver function (Total bilirubin <1.5x Upper Level of Normal (ULN); Aspartate transaminase (AST) <2.5x ULN and Alanine transaminase (ALT) <3x ULN
11. Written informed consent and ability to understand the nature of the study and the study-related procedures and to comply with them
12. Women of child-bearing potential must have a negative serum pregnancy test during screening period.

### Exclusion criteria

Patients meeting any of the following criteria are not eligible for this trial:

1. Patients with tumors of squamous, adenosquamous or other non-adenocarcinoma histology
2. Patients with advanced inoperable or metastatic esophageal adenocarcinoma
3. Esophageal adenocarcinoma staged cT1N0 and cT4b
4. Esophageal adenocarcinoma cT4a evaluated as not curatively-resectable by the local surgical investigator.
5. Gastric carcinoma (according to UICC TNM7 [15])
6. Prior chemotherapy for gastrointestinal cancer
7. Clinically-significant (active) cardiac disease (e.g. symptomatic coronary artery disease or myocardial infarction within last 12 months)
8. Clinically-significant lung disease (Forced expiratory volume in one second (FEV1) <1.5 l)
9. Peripheral neuropathy grade >1
10. Pregnant and lactating women, or patients of reproductive potential who are not using effective birth control methods. If barrier contraceptives are used, they must be continued by both sexes throughout the study.
11. Participation in another intervention trial with interference to the chemotherapeutic or chemoradiotherapeutic intervention during this study or during the last 30 days prior to informed consent.
12. Expected lack of compliance with the protocol

### Study treatment

#### Perioperative chemotherapy (Arm A)

The perioperative chemotherapy arm consists of 4 cycles of chemotherapy prior to surgery and a further 4 cycles of chemotherapy post-surgery. Each cycle of chemotherapy lasts 14 days (2 weeks). The drugs used in the FLOT regimen include 5-FU, leucovorine, oxaliplatin and docetaxel. They are applied intravenously according to the following scheme: 5-FU 2600 mg/m^2^ (24 h) day 1 and leucovorin 200 mg/m^2^ (2 h), day 1 and oxaliplatin 85 mg/m^2^ (2 h) day 1, and docetaxel 50 mg/m2 (1 h), every 2 weeks. Four neoadjuvant cycles are given over 8 weeks prior to surgery and 4 adjuvant cycles are given over 8 weeks post-surgery.

### Neoadjuvant chemoradiation (Arm B)

The neoadjuvant chemoradiation arm consists of the CROSS regimen, which includes a combination of chemotherapy and radiotherapy prior to surgery. The patient receives 5 weeks of radiation therapy and 5 concurrent weekly cycles of chemotherapy. Patients are irradiated by external beam radiation, using 3D conformal radiation technique. In detail, radiotherapy with concurrent intravenous chemotherapy is given according to the following scheme: radiotherapy with 41.4Gy given in 23 fractions of 1.8Gy: days 1–5, days 8–12, days 15–19, days 22–26 and days 29–31. Chemotherapy: paclitaxel 50 mg/m2 (1 h) day 1, 8, 15, 22, 29 and carboplatin (2 mg/ml/min AUC) (1 h) day 1, 8, 15, 22 and 29.

### Surgery (both arms)

In both arms, surgery is carried out preferably 4 to 6 weeks after the end of neoadjuvant treatment. Open, minimally-invasive or hybrid resection techniques are allowed in the trial.

### Esophageal resection

1. Esophageal adenocarcinoma with its epicenter located at the thoracic esophagus and AEG type 1 tumors are treated by subtotal esophagectomy with transthoracic resection.
2. Esophageal adenocarcinoma with its epicenter located at the cardia (AEG Type 2) are treated either by subtotal esophagectomy with transthoracic resection, transabdominal distal esophageal resection plus gastrectomy or by esophagogastrectomy, depending on both patient characteristics and local center expertise.
3. Esophageal adenocarcinoma with its epicenter located >2 cm aboral to the cardia (AEG Type 3) which are substantially infiltrating the esophagus above the Z-line are surgically treated by transabdominal distal esophageal resection plus gastrectomy.

### Extent of lymphadenectomy

In case of subtotal esophagectomy and esophagogastrectomy, a mediastinal and abdominal 2-field lymphadenectomy is carried out. In case of transhiatal resection of the distal esophagus plus gastrectomy, lower mediastinal and abdominal modified D2-lymphadenectomy is performed.

### Surgical reconstruction

After transthoracic esophagectomy, the continuity of the digestive tract is restored by a gastric tube reconstruction or colonic interposition procedure with an intrathoracic or cervical anastomosis. Reconstruction for transabdominal lower esophageal resection plus gastrectomy is carried out by esophagojejunostomy.

### Study objectives and endpoints

#### Primary endpoint

The primary endpoint is the overall survival time in the intent-to-treat population. This will be calculated as time from start of study treatment to death due to any cause. After randomization, patients will be followed up for a minimum duration of 36 months or until death. For patients alive at study closure, the survival time will be censored at time of last known survival status.

### Secondary endpoints

- Progression-free survival (PFS) time: defined in the intent-to-treat population as the time interval from randomization to the first event of locoregional failure, metastatic recurrence/progression or death. Progression is examined by computed tomography (CT) and/or upper endoscopy.
- Recurrence-free survival (RFS) time: defined in resected patients who achieved an R0 or R1 resection as the time interval from surgery to the date of first recurrence (local, regional or distant) or death, whichever comes first. Recurrence is examined by CT and/or upper endoscopy.
- Site of failure (recurrence):
  - Local failure: Patients are followed for local and regional failure irrespective of metastatic recurrence. Local failure is defined as local anastomotic or esophageal recurrence or progression, a tumor that cannot be resected or R2 resection at surgery. Local recurrence is examined by esophago-gastro-duodenoscopy.
  - Regional failure: Regional failure is defined as regional mediastinal and or celiac lymphatic recurrence or progression. Regional recurrence is examined by CT.
  - Distant failure: Distant failure is defined as the appearance of distant metastases. Subclassification of distant recurrence includes hematogenous, distant lymphatic and peritoneal/pleural recurrence. Distant recurrence is examined by CT.

Hematogenous recurrence comprises liver, lung, bone metastases and all other distant organ metastases. Distant lymphatic recurrence comprises all lymphatic metastases other than in the D1,D2 and mediastinal compartment.

- Postoperative pathologic stage:
  - Resectional status (R0/R1/R2)
  - Histo-pathological regression after neoadjuvant treatment according to Becker et al. [16]
  - Postoperative pathology according to the UICC TNM7 system [15]
- Surgical site complications:
  - Frequency of anastomotic leakage. The diagnosis of anastomotic leakage is made considering the following definition: Defect of the esophagogastric wall integrity at the anastomotic site leading to a communication between the intra- and extraluminal compartments (detection by radiographic imaging, endoscopy, on re-laparotomy or on re-thoracotomy).
  - Frequency of intrathoracic fluid collection or abscess requiring invasive treatment
  - Frequency of intraabdominal fluid collection or abscess requiring invasive treatment
  - Frequency of surgical site infection according to the Center of disease control (CDC)-definition [17]
- Non-surgical site complications:
  - Frequency of postoperative pneumonia with at least 3 of 4 of the following criteria: Purulent tracheal secretion, temperature > 37.5 °C, white blood count >12 000 or < 4500 /ml, elevated C-reactive protein level AND radiological evidence of pulmonary infection AND a positive sputum culture
  - Frequency of postoperative Acute Respiratory Distress Syndrome (ARDS), defined as severe hypoxia (PaO2/ FiO2 < 200), diffuse bilateral pulmonary infiltration and pulmonary wedge pressure less than 18 mmHg. Acute lung injury, defined as PaO2/FiO2 between 200 and 300, is considered as ARDS.
  - Frequency of postoperative major bronchial sputum with atelectasis, defined as bronchial sputum with radiographic or bronchoscopically-proven atelectasis requiring bronchoscopy and lack of fever or hyperleukocytosis
  - Frequency of postoperative respiratory failure, defined as the inability of a patient to maintain a PaO2 > 60 mmHg or a PaCO2 < 55 mmHg, requiring oro-tracheal intubation and assisted ventilation.
  - Frequency of postoperative deep venous thrombosis.
  - Frequency of postoperative lung embolism.
  - Frequency of postoperative myocardial infarction.
  - Frequency of postoperative stroke.
  - Postoperative hospital stay after surgery until discharge, in days.
  - Overall complications (Grade 2 and higher) according to the modified Clavien-Dindo classification (MCDC)
- Postoperative mortality: 30-day postoperative mortality.
- Days of hospitalization: for neoadjuvant, surgical and adjuvant treatment, in days.
- Quality of Life: measured by European Organization of Research and Treatment of Cancer (EORTC) QLQ-C30, OES18, CIPN20 questionnaire scores.

### Data collection and follow-up

#### Pre-therapeutic work-up and screening assessment

Screening evaluations have to be performed within 21 days prior to randomization. For this evaluation, inclusion and exclusion criteria are checked and validated. The complete pre-therapeutic work-up includes a physical examination, medical history, demography, vital signs, body weight, electrocardiogram, standard laboratory tests, upper endoscopy with biopsies, endoscopic ultrasound and a CT scan of the thorax and abdomen. In patients with a history or symptoms of cardiac and/or pulmonary disease, additional cardiology review/echocardiography (ejection fraction >50 %) and/or pulmonary function tests (FEV1 > 1.5 l) are mandatory. Clinical tumor staging (cTNM) is based on the data obtained from endoscopic ultrasound and CT scan. When baseline assessments are completed, check and validation of inclusion and exclusion criteria for the study is performed. Detailed information on all screening evaluations is given in Additional file 1: Table S1 and Additional file 2: Table S2.

### Assessments during the treatment phase

**Fig. 2:**
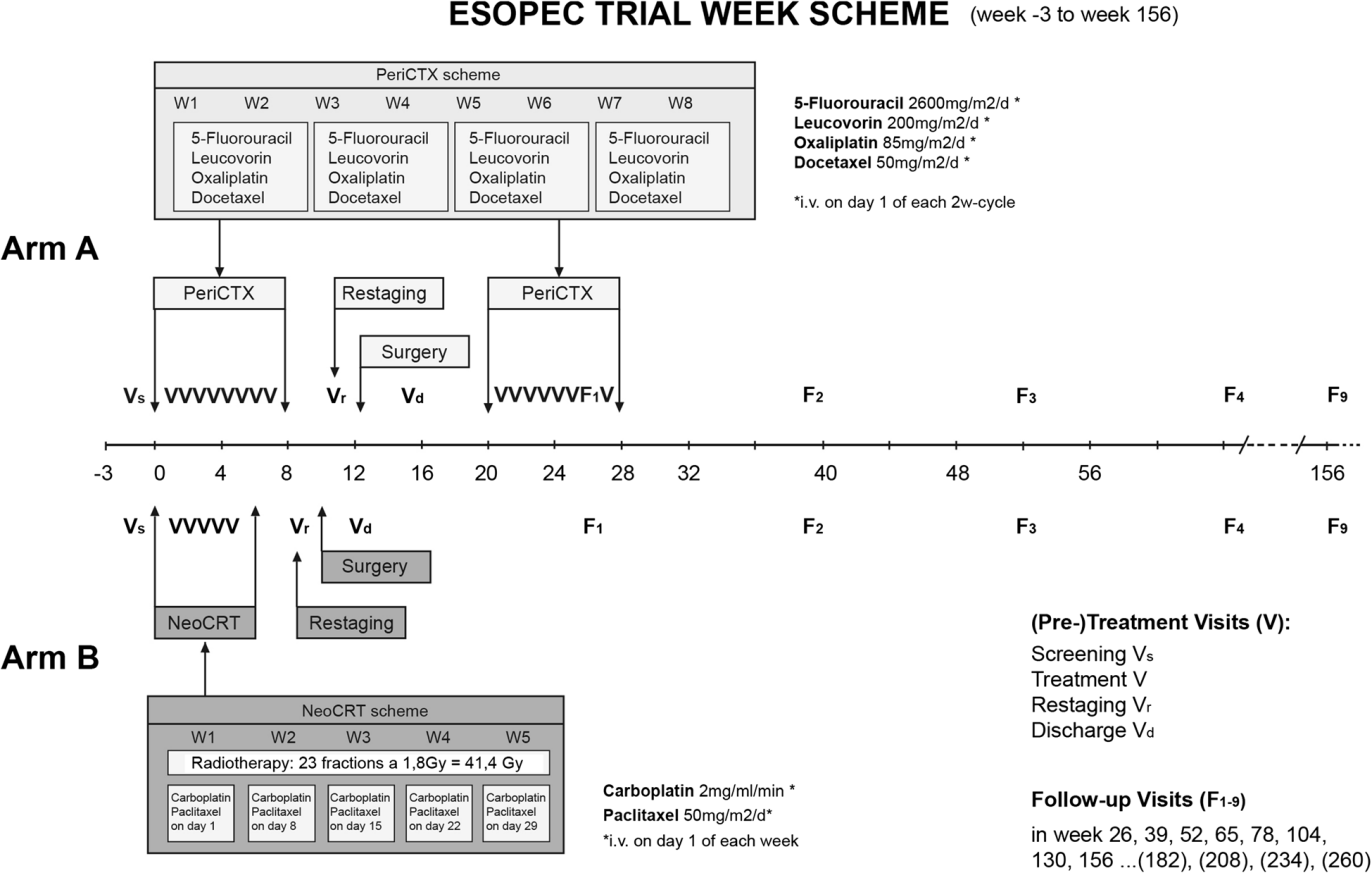
Treatment and visit week scheme of the ESOPEC trial

Treatment visits are performed biweekly during perioperative chemotherapy (Arm A) and weekly during neoadjuvant chemoradiation (Arm B) and contain measurements of patients’ vital signs, body weight, electrocardiogram and standard and hematologic laboratory tests (Fig. 2). Preoperatively, within 1–3 weeks after end of neoadjuvant treatment, clinical re-staging of the tumor is carried out by upper endoscopy and a CT scan of the thorax and abdomen. On the day of discharge from hospital after surgery, standard laboratory tests, body weight, histo-pathology report, treatment and postoperative data as well as quality of life questionnaires are assessed.

### Assessments during the follow-up phase

The first follow-up visit is performed 6 months after start of treatment, even if postoperative chemotherapy is still ongoing at that date. From then on, follow-up visits are carried out every 3 months in the first year of follow-up and every 6 months from the second year after treatment until the end of follow-up (min. 3 years) (Fig. 2). Evaluation for disease recurrence is performed by clinical visitation including physical examination, body weight and CT scan of the thorax and abdomen.

For all patients, follow-up assessment is performed until the end of the trial or death. The end the trial (end of trial follow-up) will be 3 years after the study treatment of the last patient started.

**Fig. 3:**
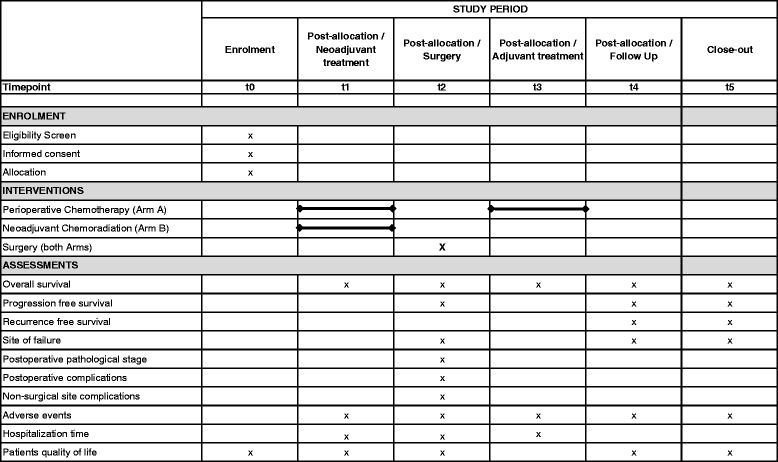
Schedule of enrolment, interventions and assessments of the ESOPEC trial

Information on the sequence of enrolment, therapeutic interventions and outcome assessments is given in Fig. 3. Detailed information on all assessments during the pre-therapeutic phase, treatment phase and follow-up phase are given in Additional file 1: Table S1 and Additional file 2: Table S2.

### Statistical planning

The sample size of the ESOPEC trial has been planned to ensure sufficient power to demonstrate an overall survival advantage of perioperative chemotherapy compared to neoadjuvant chemoradiation. The sample size calculation is based on the primary endpoint overall survival. It is assumed that the overall survival rate for esophageal adenocarcinoma patients treated with neoadjuvant chemoradiation according to the CROSS regimen is 55 % at 3 years after randomization, as observed in a comparable setting [3]. For patients with perioperative (neoadjuvant and adjuvant) chemotherapy according to the FLOT regimen, a conservative assumption of an overall survival rate of 68 % is made. This assumption is based on survival rates of about 72 % reported for the treatment based on FLOT (i.e. FLOT + surgery + FLOT) in a different patient population [8] as well as our own experience of 68 % [10] and 70 % 3-year overall survival (retrospective analysis; esophageal adenocarcinoma; all patients treated with FLOT + surgery + FLOT; unpublished data). This corresponds to a hazard ratio of 0.645 of treatment arm A compared to treatment arm B. The treatment effect will be assessed by estimation of the hazard ratio with corresponding asymptotic two-sided 95 % confidence interval. To test superiority of arm A (based on FLOT) over arm B (based on CROSS) at one-sided significance level of 2.5 %, the null hypothesis is rejected if the asymptotic two-sided 95 % confidence interval lies completely below one. Under the above assumptions, the study is planned to detect superiority of arm A over arm B with a power of 80 %, which requires a total number of 163 events (deaths) to be observed. Yet, only 88-97 % of randomized patients are expected to be resectable at the time of surgery due to deterioration of tumor staging or the patient’s overall condition [3, 7]. To account for a possibly diminished observed hazard ratio, the sample size is calculated to achieve a power of 90 %. Therefore, 218 events (deaths) have to be observed. The required number of patients to be randomized to observe this number of events depends on the length of follow-up. With a recruitment period of 3 years and an additional follow-up period after the end of recruitment of 3 years, it can safely be assumed that a sufficient number of events will have been observed by the end of the trial if a total of 438 patients are available for analysis.

The effect of treatment with respect to the primary endpoint overall survival will be estimated and tested by Cox regression. The regression model will include treatment (Arm A: FLOT vs. ArmB: CROSS), N stage (N0, N+), age and trial center as independent variables. As estimate of the effect size, the hazard ratio between the two treatment arms will be given with the corresponding asymptotic two-sided 95 % confidence interval. The two-sided test on the difference between the two treatment arms at two-sided significance level 5 % will be based on the corresponding asymptotic two-sided 95 % confidence interval from the Cox regression model. Overall survival rates in the two treatment arms will be estimated by the Kaplan-Meier method.

### Translational substudies

In the setting of the ESOPEC trial, correlative substudies are included addressing the value of circulating tumor cells, circulating tumor DNA, circulating miRNA, adipokines, inflammatory markers and possible proteomic determinants of malignancy as biomarkers for prognosis and treatment outcomes. Therefore, patient blood samples are drawn and analyzed pretherapeutically and during the course of treatment. Formalin-fixed paraffin-embedded (FFPE) tumor specimens are centrally biobanked and analyzed. Tumor specimens include material from pre-treatment biopsies and operative specimens. A tissue bank of esophageal adenocarcinoma treated in well- standardized protocols with highly-controlled prospective clinical follow-up data will be created. The biological substudies of the trial are designed for validation of existing hypotheses and for discovery and generation of new hypotheses addressing important questions in chemotherapeutic and radiotherapeutic research of esophageal adenocarcinoma. Specifically, focus is on questions concerning biological determinants of chemotherapeutic and radiotherapeutic response and resistance. Comparison of pretherapeutic and intra- and posttherapeutic samples and specimens by the methods mentioned above may disclose biological differences in the tumors which could be used as criteria for choice of a specific therapeutic protocol in the future.

## Discussion

The prognosis of surgically-treated esophageal adenocarcinoma has progressively improved over the past 20 years [18, 19]. Besides improvement of patient selection, preoperative staging, perioperative critical care and surgical technique, this improved outcome is attributed to the incremental inclusion of patients with esophageal adenocarcinoma in multimodal treatment protocols [18–20]. Since the publication of the MAGIC, ACCORD-07 and CROSS trials in the years 2006 – 2012, these protocols have broadly entered clinical practice in Europe and have been integrated in national and international guidelines for the treatment of locally-advanced esophageal adenocarcinoma [2, 3, 6, 7]. Although different studies have been carried out comparing either neoadjuvant chemoradiation or perioperative chemotherapy plus surgery versus surgery alone for esophageal and/or gastric adenocarcinoma, no prospective data comparing the contemporary regimens of neoadjuvant chemoradiation and perioperative chemotherapy in patients with esophageal adenocarcinoma are available. The present trial was started to obtain valid information whether neoadjuvant chemoradiation or perioperative chemotherapy yields superior benefits for the curative treatment of esophageal adenocarcinoma.

The ESOPEC trial compares perioperative chemotherapy plus surgery with neoadjuvant chemoradiation plus surgery, which serves as the control. Neoadjuvant chemoradiation plus surgery is the most widely-established treatment modality for esophageal cancer in Germany and some other Western countries and has been practiced for more than two decades. As neoadjuvant chemoradiation plus surgery has already been proven by an RCT [3] to be superior to surgery alone, it serves as the control group and no surgery alone group is added. The neoadjuvant chemoradiation protocol with 41.4Gy plus carboplatin/paclitaxel (CROSS protocol) was chosen as the control intervention in the proposed trial, as it is the protocol with the highest evidence for prolongation of survival since the publication of the CROSS trial in 2012 [4]. Moreover, the CROSS protocol offers the benefit of moderate toxicity compared to previously-used regimens [4, 21]. For these reasons, the CROSS protocol has become widely distributed not only in the recruiting centers of this trial but also internationally in Western Europe and North America.

Perioperative chemotherapy plus surgery has been used frequently in specialized German cancer centers. The FLOT regimen has been chosen for the experimental arm because there are indications of superior response compared to previously-used taxane-free chemotherapy protocols, especially in patients with esophageal adenocarcinoma [8, 13], offering the chance for achieving higher R0 resection rates and better control of occult distant metastases. Although no results from RCTs and/or phase III studies have been published for perioperative chemotherapy with FLOT thus far, the promising results from phase II have made FLOT the protocol of choice for perioperative treatment of esophageal adenocarcinoma and gastric cancer in many specialized German cancer centers.

## Conclusion

The ESOPEC trial is a multicenter prospectively-randomized controlled trial, comparing neoadjuvant chemoradiation according to the CROSS regimen followed by surgery versus perioperative chemotherapy according to the FLOT regimen and surgery for the curative treatment of esophageal adenocarcinoma. It is hypothesized that perioperative chemotherapy will result in increased overall survival due to a comparable local effect and a better control of micrometastatic distant disease.

## Abbreviations

AEG, esophagogastric adenocarcinoma; ALT, alanine transaminase; ARDS, acute respiratory distress syndrome; AST, aspartate transaminase; AUC, area under the curve; CDC, center of disease control; CROSS protocol, “Chemoradiation followed by surgery versus surgery alone for patients with adenocarcinoma or squamous cell carcinoma of the esophagus”; CT, computed tomography; ECF, epirubricine-cisplatin-5FU; ECOG, Eastern cooperative oncology group; ECX, epirubricine-cisplatin-capecitabine; EORTC, European Organization of research and treatment of cancer; FEV1, forced expiratory volume in one second; FFPE, formalin-fixed paraffin-embedded; FLOT protocol, 5-FU/leucovorin/oxaliplatin/docetaxel; Gy, Grey; MCDC, modified clavien-dindo classification; PFS, progression-free survival; RCT, randomized controlled trial; RFS, recurrence-free survival; UICC, Union internationale contre le cancer; ULN, upper level of normal

## Additional files

Additional file 1: Table S1. Visit schedule Arm A (FLOT). (DOCX 24 kb) Additional file 2 Table S2. Visit schedule Arm B (CROSS). (DOCX 25 kb) Additional file 3: Participating centers (July 2016). (DOCX 15 kb)
