# Supplementary material for: ESOPEC: prospective randomized controlled multicenter phase III trial comparing perioperative chemotherapy (FLOT protocol) to neoadjuvant chemoradiation (CROSS protocol) in patients with adenocarcinoma of the esophagus (NCT02509286)
Source: BMC Cancer. 2016 Jul 19;16:503. doi: 10.1186/s12885-016-2564-y (PMC4952147; doi:10.1186/s12885-016-2564-y)
Supplement: Additional file 1: Table S1. — Visit schedule Arm A (FLOT). (DOCX 24 kb) [file 12885_2016_2564_MOESM1_ESM.docx]

**Table 1.** Visit schedule Arm A

| **Visit Name** | **Screening** | **Treatment preoperative** | **Preoperative** | **Discharge** | **Treatment post-operative** | **Follow-up** |
| --- | --- | --- | --- | --- | --- | --- |
| Timepoint | <21 days before rando-misation | Week 1-8  At day 1 of each chemo-therapeutic cycle | Within 1-3 weeks after end of neoadjuvant treatment | Day of hospital  discharge from Surgery | Beginning 4-6 weeks after discharge.  At day 1 of each chemo-therapeutic cycle | Beginning in Week 26  Every  3/6 months^[[1]](#endnote-1)^*  **(±14 days)** |
|  | **(Vs)** | **(V)** | **(Vr)** | **(Vd)** | **(V)** | **(F1--x)** |
| Informed consent | x |  |  |  |  |  |
| Inclusion/Exclusion criteria | x |  |  |  |  |  |
| Demography | x |  |  |  |  |  |
| Medical history | x |  |  |  |  |  |
| Vital signs (P, RR, T) | x | x | x |  | x |  |
| Physical examination^[[2]](#endnote-2)^ | x |  | x |  |  | x |
| Body weight | x | x | x | x | x | x |
| ECOG performance score | x |  |  |  |  |  |
| CT Thorax / Abdomen | x^[[3]](#endnote-3)^ |  | x |  |  | x^[[4]](#endnote-4)^ |
| Endoscopic ultrasound^[[5]](#endnote-5)^ | x |  |  |  |  |  |
| Upper GI endoscopy | x^II^ |  | x |  |  |  |
| Histo-pathology (report) | x |  |  | x |  |  |
| Tissue (paraffin embedded block)^[[6]](#endnote-6)^ | x |  |  | x |  |  |
| ECG^[[7]](#endnote-7)^ | x |  | x |  |  |  |
| Laboratory hematology and chemistry^[[8]](#endnote-8)^ | x | x | x | x | x |  |
| Laboratory coagulation^[[9]](#endnote-9)^ | x |  | x |  |  |  |
| Pregnancy test^[[10]](#endnote-10)^ (only women of childbearing potential) | x |  |  |  |  |  |
| Randomization | x |  |  |  |  |  |
| Treatment data |  | x |  | x | x |  |
| Postoperative data^[[11]](#endnote-11)^ |  |  |  | x |  |  |
| Blood sample for translational research |  | x^[[12]](#endnote-12)^ | x | x | - - - - 1. x^19^ |  |
| Adverse events and complications^[[13]](#endnote-13)^ | x | | | | | |
| Concomitant medication^[[14]](#endnote-14)^ | x | | | | |  |
| Quality of life (EORTC QLQ-C30, OES18, CIPN20) | x |  | x | x |  | x |

1. * The first follow-up visit is performed 6 months after start of treatment, even if postoperative chemotherapy is still ongoing at that date. From then on, follow-up visits are carried out every 3 months (+/- 7days) in the first year of follow-up and every 6 months (+/- 7days) from the second year after treatment until the end of follow-up (min. 3 years). [↑](#endnote-ref-1)
2. Thorough physical/medical examination includes, but is not limited to, cardiovascular, gastrointestinal, hepatobiliary, respiratory, musculoskeletal, skin, neurological, genitourinary/renal and other organ systems. Physical examination incl. body height at screening. [↑](#endnote-ref-2)
3. Not older than 28 days before date of randomisation. [↑](#endnote-ref-3)
4. CT Thorax/Abdomen is carried out regularly at follow-up visits. Further diagnostic investigations other than laboratory and CT (PET-CT, Upper endoscopy) are performed only when clinically indicated at the discretion of the investigator/treating physician. [↑](#endnote-ref-4)
5. Endoscopic ultrasound is carried out if applicable. In case of technical non-applicability in highly-obstructing tumors, the pre-therapeutic cT and cN stages will be determined in CT Thorax/Abdomen. [↑](#endnote-ref-5)
6. Representative paraffin blocks from the initial biopsy and the operative specimen will be requested from the reporting pathologists. [↑](#endnote-ref-6)
7. ECG must be performed during screening visit and preoperative. Patients with a cardiac history should have an additional cardiology review and should have a left ventricular ejection fraction > 50% as determined by echocardiography. [↑](#endnote-ref-7)
8. ^VII^ Hematology includes hemoglobin, total white blood count, platelet count, and a differential white count including neutrophils, lymphocytes, monocytes, eosinophils and basophils. Biochemistry includes (but is not limited to) sodium, potassium, serum creatinine, AST, ALT, total bilirubin and blood glucose. [↑](#endnote-ref-8)
9. Coagulation includes PTT and INR. [↑](#endnote-ref-9)
10. Not older than +/- 7 days [↑](#endnote-ref-10)
11. Including data on surgical procedure and pathology. [↑](#endnote-ref-11)
12. Blood samples for translational research during neoadjuvant chemotherapy are only collected once at day 1 of the first neoadjuvant treatment cycle. [↑](#endnote-ref-12)
13. The AE reporting period for this trial begins after first intake of medication within the study until 8 months after randomisation. All adverse events have to be documented in the CRF. [↑](#endnote-ref-13)
14. Concomitant medication must be available in the source data and don`t be captured in the CRF. [↑](#endnote-ref-14)
