# Supplementary material for: ESOPEC: prospective randomized controlled multicenter phase III trial comparing perioperative chemotherapy (FLOT protocol) to neoadjuvant chemoradiation (CROSS protocol) in patients with adenocarcinoma of the esophagus (NCT02509286)
Source: BMC Cancer. 2016 Jul 19;16:503. doi: 10.1186/s12885-016-2564-y (PMC4952147; doi:10.1186/s12885-016-2564-y)
Supplement: Additional file 3: — Participating centers (July 2016). (DOCX 15 kb) [file 12885_2016_2564_MOESM3_ESM.docx]

**The following 16 participating centers of the ESOPEC trial and have been approved by their local ethics committees and by the leading ethics committee at Albert-Ludwigs-University Freiburg/Germany.**

University of Schleswig-Holstein Kiel – Medical Center

University of Schleswig-Holstein Lübeck– Medical Center

University of Hamburg – Medical Center

Charite Berlin – Medical Center

University of Münster – Medical Center

University of Düsseldorf – Medical Center

University of Magdeburg – Medical Center

Technical University of Dresden – Medical Center

RTWH University of Aachen – Medical Center

University of Leipzig – Medical Center

Sana Medical Center Offenbach

University of Mainz – Medical Center

University of Würzburg – Medical Center

LMU University of Munich – Medical Center

University of Göttingen – Medical Center

Albert-Ludwigs University of Freiburg – Medical Center
